# *KLRD1*-expressing natural killer cells predict influenza susceptibility

**DOI:** 10.1186/s13073-018-0554-1

**Published:** 2018-06-14

**Authors:** Erika Bongen, Francesco Vallania, Paul J. Utz, Purvesh Khatri

**Affiliations:** 1 Institute for Immunity, Transplantation and Infection, Stanford University School of Medicine, Stanford, CA 94305 USA; 2 Program in Immunology, Stanford University School of Medicine, Stanford, 94305 CA USA; 3 Department of Medicine, Division of Biomedical Informatics Research, Stanford University School of Medicine, Stanford, CA 94305 USA; 4 Department of Medicine, Division of Immunology and Rheumatology, Stanford University School of Medicine, Stanford, CA 94305 USA

**Keywords:** Influenza, Natural killer cells, Hematopoietic stem and progenitor cells, KLRD1, CD94

## Abstract

**Background:**

Influenza infects tens of millions of people every year in the USA. Other than notable risk groups, such as children and the elderly, it is difficult to predict what subpopulations are at higher risk of infection. Viral challenge studies, where healthy human volunteers are inoculated with live influenza virus, provide a unique opportunity to study infection susceptibility. Biomarkers predicting influenza susceptibility would be useful for identifying risk groups and designing vaccines.

**Methods:**

We applied cell mixture deconvolution to estimate immune cell proportions from whole blood transcriptome data in four independent influenza challenge studies. We compared immune cell proportions in the blood between symptomatic shedders and asymptomatic nonshedders across three discovery cohorts prior to influenza inoculation and tested results in a held-out validation challenge cohort.

**Results:**

Natural killer (NK) cells were significantly lower in symptomatic shedders at baseline in both discovery and validation cohorts. Hematopoietic stem and progenitor cells (HSPCs) were higher in symptomatic shedders at baseline in discovery cohorts. Although the HSPCs were higher in symptomatic shedders in the validation cohort, the increase was statistically nonsignificant. We observed that a gene associated with NK cells, *KLRD1*, which encodes CD94, was expressed at lower levels in symptomatic shedders at baseline in discovery and validation cohorts. *KLRD1* expression in the blood at baseline negatively correlated with influenza infection symptom severity. *KLRD1* expression 8 h post-infection in the nasal epithelium from a rhinovirus challenge study also negatively correlated with symptom severity.

**Conclusions:**

We identified *KLRD1*-expressing NK cells as a potential biomarker for influenza susceptibility. Expression of *KLRD1* was inversely correlated with symptom severity. Our results support a model where an early response by *KLRD1*-expressing NK cells may control influenza infection.

**Electronic supplementary material:**

The online version of this article (10.1186/s13073-018-0554-1) contains supplementary material, which is available to authorized users.

## Background

Influenza is a major public health problem that causes 9 to 35 million illnesses annually in the USA [1]. Children, older adults, pregnant women, and immunocompromised patients are at an increased risk of influenza infection. Within healthy young adults, influenza susceptibility is difficult to predict as responses to influenza exposure vary from no detectable infection to severe disease. A better understanding of the immune determinants of influenza susceptibility is necessary to identify novel high-risk populations and design better vaccines.

Human influenza challenge studies provide a unique opportunity to study influenza susceptibility. In these studies, healthy individuals are inoculated with live influenza virus, and viral shedding titers and self-reported symptom scores are measured over the course of infection. Infected individuals fall into four groups: symptomatic shedders, asymptomatic nonshedders, symptomatic nonshedders, and asymptomatic shedders. Previous challenge studies have used transcriptional data to distinguish symptomatic shedders from asymptomatic nonshedders post-infection [2], detect infection prior to symptom onset [3], develop transcriptional signatures of symptom status [4, 5], and prototype individualized predictors for infection [6]. However, to our knowledge, no cellular or transcriptional signatures that can predict infection susceptibility prior to inoculation have been reported.

Relatively little work has been done examining how preexisting immune cell populations affect influenza susceptibility. Wilkinson et al. demonstrated in an H3N2 influenza challenge study that higher baseline levels of influenza-specific CD4+ T cells in the blood were associated with reduced viral shedding and less severe symptoms [7]. Sridhar et al. followed healthy adults during two consecutive flu seasons and found that adults with higher baseline levels of influenza-specific CD8+ T cells experienced lower symptom severity [8]. To our knowledge, the role of immune cell frequencies in influenza susceptibility beyond the T cell compartment has not been described.

Cell mixture deconvolution is an established computational approach to estimate immune cell proportions from bulk tissue gene expression data, either from blood or solid tissue [9]. The key assumption of cell mixture deconvolution is that the gene expression of a bulk tissue sample can be explained by the underlying ratio of cell types and the expression profiles of those cell types. Deconvolution methods define specific cell types using a reference matrix, known as a basis matrix, of expected cell type expression. The basis matrix is used by an algorithm, such as linear regression, to predict the proportion of each cell type in bulk tissue samples. Cell mixture deconvolution has been used to profile the immune response to leprosy and across cancers [10, 11]. We have described a deconvolution basis matrix, immunoStates, that accurately estimates cellular proportions for 20 immune cell subsets by reducing biological, methodological, and technical biases [12]. In this study, we used the immunoStates basis matrix with a linear regression model.

We hypothesized that immune cell populations at baseline (i.e., prior to exposure to influenza) may affect influenza susceptibility. To test this hypothesis, we used 4 influenza challenge studies (3 discovery, 1 validation) composed of 52 samples (40 discovery, 12 validation). We estimated proportions of 20 immune cell subsets in each sample using the immunoStates matrix and a linear regression model. We performed a multi-cohort analysis of estimated immune cell proportions between symptomatic shedders and asymptomatic nonshedders at baseline across the three discovery influenza challenge studies. Symptomatic shedders had lower proportions of natural killer (NK) cells at baseline in discovery cohorts and the held-out validation cohort. Symptomatic shedders had significantly higher proportions of hematopoietic stem and progenitor cells (HSPCs) at baseline. Although the validation cohort demonstrated the same trend, it was not statistically significant. NK cell-associated gene *KLRD1* expression was also significantly lower in the blood of symptomatic shedders at baseline in discovery and validation cohorts and correlated negatively with symptom severity. Increased *KLRD1* expression may be associated with increased proportions of cytotoxic cells, as *KLRD1* expression at baseline correlated with cytotoxic granule-associated genes *CCL5*, perforin (*PRF1*), and several granzymes (*GZMA*, *GZMB*, and *GZMH*). We also observed that *KLRD1* expression decreased in the blood during the first 48 h of influenza infection. We examined *KLRD1* expression in the nasal epithelium in human rhinovirus (HRV) and respiratory syncytial virus (RSV) infection as robust common immune response across these viruses has been described [13]. *KLRD1* expression significantly increased in nasal epithelium during infection with HRV or RSV. In an HRV challenge cohort, symptom severity correlated negatively with expression of *KLRD1* in the nasal epithelium 8 h post-infection. This data supports a model where a rapid antiviral response by *KLRD1*-expressing NK cells may control viral infection.

## Methods

### Identification and preprocessing of cohorts

We identified 4 influenza challenge studies consisting of 52 whole blood samples from the NCBI database Gene Expression Omnibus (GEO) (Table 1). We supplemented the influenza challenge cohorts with 7 acute viral infection studies consisting of 16 cohorts of 771 whole blood, PBMC, and nasal epithelium samples from GEO (Table 2) [14]. We excluded challenge studies with less than five asymptomatic nonshedders or five symptomatic shedders. We used phenotypic labels as reported by the original authors. All datasets used were publicly available (Additional file 1: Supplemental Methods).

**Table 1:** Influenza challenge cohorts

Cohort	Group	Virus	Tissue	Asymptomatic nonshedders	Symptomatic shedders	Platform	Citations
GSE73072 challenge A	Discovery challenge	H1N1	Whole blood	6	8	Affymetrix	[2, 3, 6]
GSE73072 challenge B	Discovery challenge	H3N2	Whole blood	6	7	Affymetrix	[2, 3, 6]
GSE73072 challenge C	Discovery challenge	H3N2	Whole blood	6	7	Affymetrix	[6]
GSE61754	Validation challenge	H3N2	Whole blood	5	7	Illumina	[5]
Total		2	1	23	29	2

**Table 2:** Additional viral infection cohorts

Cohort	Virus	Tissue	Controls	Acute infection	Platform	Citations
GSE11348	HRV	Nasal scrapings	15	15	Affymetrix	[22]
GSE97742 HRV	HRV	Nasopharyngeal swabs	30	30	Illumina	[23]
GSE97742 RSV	RSV	Nasopharyngeal swabs	38	38	Illumina	[23]
GSE97742 RSVco	RSV co-infected with other viruses	Nasopharyngeal swabs	15	15	Illumina	[23]
GSE61821 mild H1N1	Seasonal H1N1	Whole blood	36	32	Illumina	[40]
GSE61821 mild H3N2	Seasonal H3N2	Whole blood	13	15	Illumina	[40]
GSE61821 severe H1N1	Seasonal H1N1	Whole blood	19	16	Illumina	[40]
GSE61821 severe H3N2	Seasonal H3N2	Whole blood	6	7	Illumina	[40]
GSE61821 pandemic H1N1	Pandemic H1N1	Whole blood	8	10	Illumina	[40]
GSE68310 flu	Influenza A	Whole blood	40	34	Illumina	[24]
GSE43777	Dengue	PBMC	45	45	Affymetrix	[41]
GSE51808	Dengue	Whole blood	9	13	Affymetrix	[42]
GSE68310 HRV	HRV	Whole blood	20	20	Illumina	[24]
GSE97741 HRV	HRV	Whole blood	25	24	Illumina	[23]
GSE67059 RSV	RSV	Whole blood	20	65	Illumina	[43]
GSE97741 RSV	RSV	Whole blood	25	28	Illumina	[23]
Total	8	4	364	407		

### Cell mixture deconvolution using immunoStates

We performed cell mixture deconvolution using the immunoStates basis matrix and a linear regression model, as described previously, to estimate the immune cell frequencies for 20 immune cell subsets in blood or nasal epithelium gene expression data [12]. We removed all cell types that were not detected in any samples (Additional file 1: Table S1). If a cell type was detected in a subset of samples, values of zero were set to an arbitrarily low number and each sample was rescaled so that the cell type proportions summed to 100% in each sample.

### Integrated multi-cohort analysis of cellular proportions

We performed an integrated multi-cohort analysis using the MetaIntegrator R package [15]. To analyze differences in cell proportions, we utilized random effects inverse variance model-based meta-analysis by combining effect sizes, as described previously [13, 15, 16]. We estimated the change in proportion for each cell type in each cohort between symptomatic shedders and asymptomatic nonshedders as Hedge’s adjusted *g*. We combined the changes in cellular proportion for each cell type into a summary effect size using a linear combination of study-specific effect sizes, where each cohort-specific effect size was weighted by the inverse of that cohort’s pooled variance [15, 17]. We performed multiple hypotheses testing correction using the Benjamini-Hochberg false discovery rate (FDR) [18].

## Results

### Dataset description

**Fig. 1:**
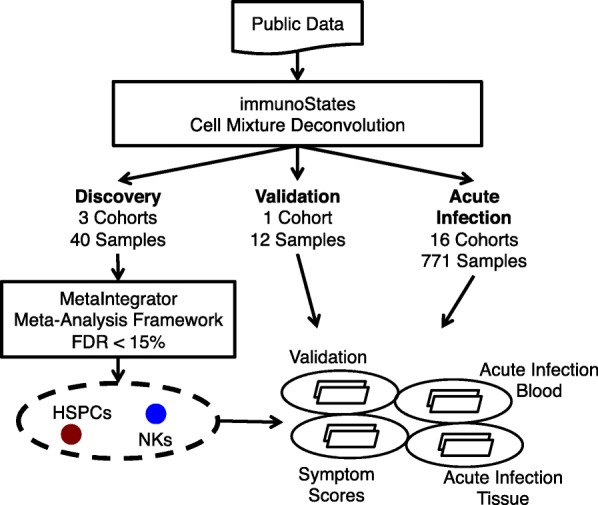
Schematic of experimental design. From NCBI GEO, we identified gene expression microarray datasets from influenza viral challenge studies and studies of naturally acquired infection. Using immunoStates cell mixture deconvolution, we estimated the immune cell proportions in each sample. We separated the challenge studies into discovery and validation cohorts. We analyzed differences at baseline between symptomatic shedders and asymptomatic nonshedders using MetaIntegrator, a meta-analysis framework. We identified immune cell types with significantly different proportions between symptomatic shedders and asymptomatic nonshedders, which we further investigated in an influenza challenge validation cohort, and cohorts of naturally acquired acute infections

We identified four human influenza challenge studies from the NCBI database Gene Expression Omnibus (GEO) (Table 1 and Fig. 1) [14]. Each of these studies profiled the whole blood transcriptome of healthy individuals inoculated with live H1N1 or H3N2 influenza at baseline and the subsequent 2–7 days. These studies defined viral shedding status based on influenza laboratory tests and symptom status based on self-reported modified Jackson scores [5, 6].

We chose three of the challenge cohorts as discovery cohorts as they were part of a single study and all profiled samples using Affymetrix microarrays [6]. The remaining dataset, GSE61754, profiled samples using Illumina microarrays and was used as a validation cohort [5]. This choice allowed us to ensure that our deconvolution analysis was robust to the microarray platform used. We only included baseline samples from subjects with concordant symptom and shedding status (symptomatic shedders and asymptomatic nonshedders).

### Integrated multi-cohort analysis of estimated cell proportions

**Fig. 2:**
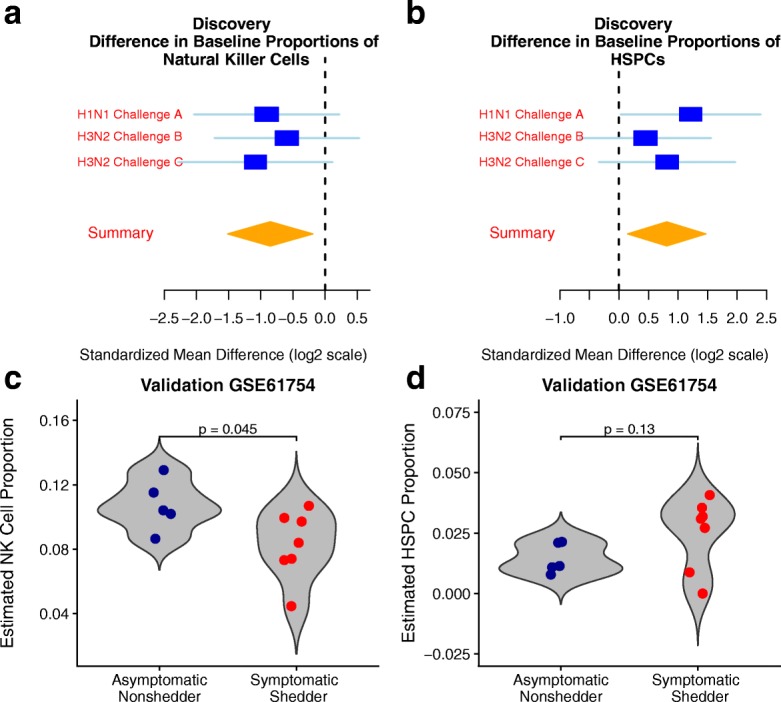
Differences in estimated cell type proportions between asymptomatic nonshedders and symptomatic shedders before infection. Immune cell proportions were estimated at baseline using cell mixture deconvolution. Forest plots of effect sizes of **a** NK cells (effect size = − 0.85, *P* = 0.012) and **b** HSPCs (effect size = 0.81, *P* = 0.017) in discovery cohorts. Positive effect sizes indicate higher levels while negative effect sizes indicate lower levels for that cell type in symptomatic shedders. The *x* axes represent standardized mean difference between symptomatic shedders and asymptomatic nonshedders, computed as Hedges’ *g*, in log2 scale. The size of the blue rectangles is proportional to the SEM difference in the study. Whiskers represent the 95% confidence interval. The yellow diamonds represent overall, combined mean difference for a given cell type. Width of the yellow diamonds represents the 95% confidence interval of overall mean difference. Violin plots of estimated cell proportions of **c** NK cells (effect size = − 1.18, *P* = 0.045) and **d** HSPCs (effect size = 0.79, *P* = 0.13) at baseline in validation cohort GSE61754. NK, natural killer. HSPC, hematopoietic stem and progenitor cells

We hypothesized that the immune cell profiles of symptomatic shedders and asymptomatic nonshedders would be different prior to inoculation. To test this hypothesis, we estimated proportions of 20 immune cell types in each sample in each cohort using immunoStates and a linear regression model [12]. We removed 8 out of 20 cell types from further analysis as they were not detected in at least one dataset (Additional file 1: Table S1). A multi-cohort analysis of estimated cellular proportions for the remaining cell types in discovery cohorts using MetaIntegrator found that proportions of NK cells were significantly lower (*P* = 0.012, FDR < 15%; Fig. 2a), and hematopoietic stem and progenitor cells (HSPCs) were significantly higher (*P* = 0.017, FDR < 15%; Fig. 2b) in symptomatic shedders at baseline. We also observed significantly lower NK cell proportions at baseline in symptomatic shedders in the validation cohort (*P* = 0.045; Fig. 2c). Although the validation cohort exhibited a trend of higher proportions of HSPCs in symptomatic shedders at baseline, this increase was not statistically significant (*P* = 0.13; Fig. 2d).

### Identification of *KLRD1* as an NK cell-associated gene relevant to influenza challenge

**Fig. 3:**
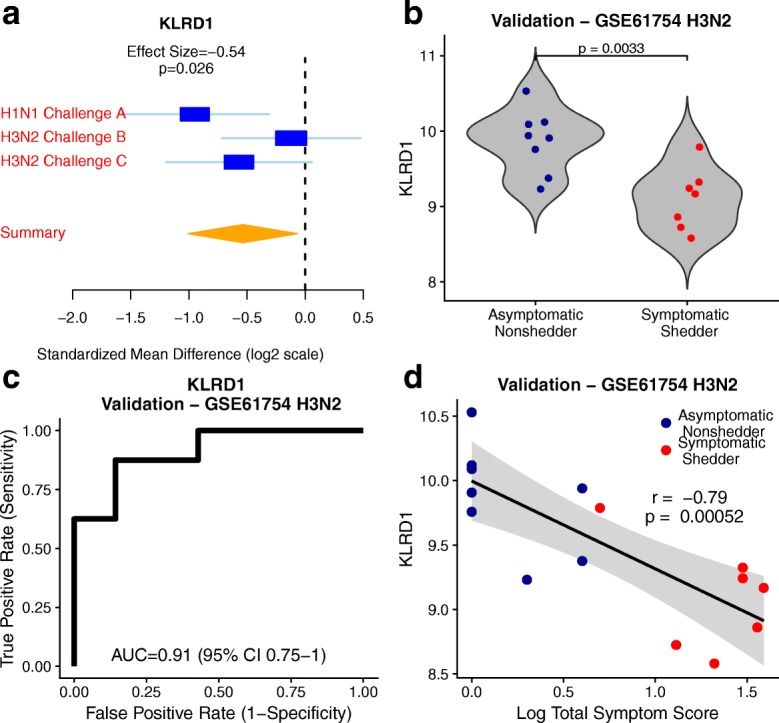
CD94 encoding gene *KLRD1* is differentially expressed between asymptomatic nonshedders and symptomatic shedders and correlates with symptom severity at baseline. **a** Forest plot of effect sizes of baseline *KLRD1* expression in discovery cohorts (summary effect size = − 0.54, *P* = 0.026). The *x* axes represent standardized mean difference between symptomatic shedders and asymptomatic nonshedders, computed as Hedges’ *g*, in log2 scale. The size of the blue rectangles is proportional to the SEM difference in the study. Whiskers represent the 95% confidence interval. The yellow diamonds represent overall, combined mean difference for a given gene. Width of the yellow diamonds represents the 95% confidence interval of overall mean difference. **b** Violin plot of *KLRD1* expression at baseline in validation cohort GSE61754 (*P* = 0.0033). **c** ROC plot of performance of *KLRD1* expression to differentiate asymptomatic nonshedders and symptomatic shedders at baseline (AUC = 0.91, 95% CI 0.75–1.0). **d** Correlation between baseline *KLRD1* expression and logged total symptom score in validation cohort GSE61754 (*r* = − 0.79, *p* = 0.00052)

A basis matrix in deconvolution defines a set of genes as a proxy for the presence of a cell type in a sample. Therefore, a significant reduction in NK cell proportions suggests that a subset of genes in immunoStates representing NK cells should be downregulated at baseline in symptomatic shedders compared to asymptomatic nonshedders. One of the 19 NK cell-related genes in immunoStates, *KLRD1*, was significantly downregulated in symptomatic shedders in discovery cohorts (summary ES = − 0.54, *P* = 0.026; Fig. 3a) and the validation cohort (*P* = 3.3e−3; Fig. 3b). In a validation cohort, *KLRD1* expression in the blood prior to infection differentiated between symptomatic shedders and asymptomatic nonshedders with high accuracy (AUROC = 0.91, 95% CI 0.75–1.0; Fig. 3c). Interestingly, the baseline expression of *KLRD1* was significantly inversely correlated with total symptom scores (*r* = − 0.79, *P* = 5.2e−4; Fig. 3d) in the validation cohort and was marginally significant (*r* = − 0.48, *P* = 0.07) in one of the two discovery cohorts where total symptom scores were available (Additional file 1: Figure S1). This suggests that *KLRD1*-expressing NK cells may be important for controlling influenza symptom severity.

### *KLRD1* baseline expression correlates with *KLRC3* and cytotoxic granule associated genes

**Fig. 4:**
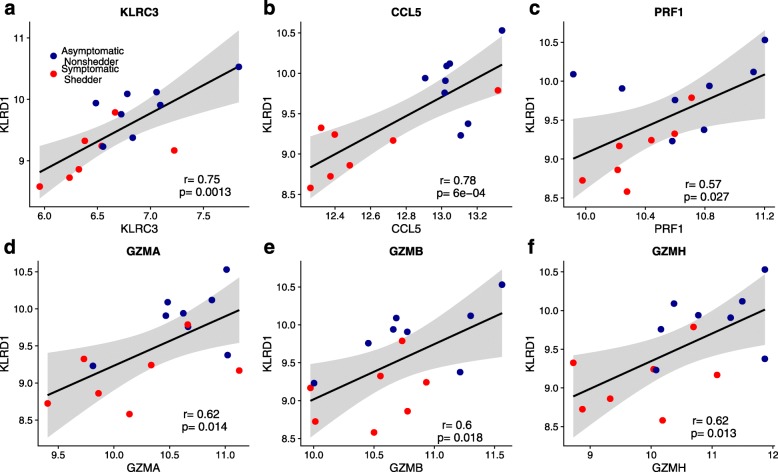
*KLRD1* correlates with *KLRC3*, and cytotoxic granule-associated genes before infection. Gene expression from validation cohort GSE61754 prior to infection demonstrating correlations between *KLRD1* expression and **a** *KLRC3* (*r* = 0.75, *P* = 0.0013) and **b**–**f** cytotoxic granule-associated genes: *CCL5* (*r* = 0.78, *P* = 0.0006), perforin (*PRF1*, *r* = 0.57, *P* = 0.027), granzyme A (*GZMA*, *r* = 0.62, *P* = 0.014), granzyme B (*GZMB*, *r* = 0.6, *P* = 0.018), and granzyme H (*GZMH*, *r* = 0.62, *P* = 0.013)

*KLRD1* encodes NK cell receptor CD94 that forms a heterodimer with several *NKG2* family members [19]. To determine whether *KLRD1* expression was associated with a particular *NKG2* family member, we correlated *KLRD1* expression at baseline with three *NKG2* family member encoding genes: *KLRC1*, *KLRC2*, and *KLRC3*. Only *KLRC3*, which encodes protein isoforms NKG2E and NKG2H, significantly correlated with *KLRD1* in the validation cohort (*r* = 0.75, *P* = 1.3e−3; Fig. 4a) and discovery cohorts (*r* = 0.4, *P* = 7.1e−3; Additional file 1: Figure S2a).

To determine whether expression of *KLRD1* was associated with a cytotoxic transcriptional signature, we correlated expression of *KLRD1* at baseline with genes associated with cytotoxic granules. While releasing cytotoxic granules, NK cells also release CCL5 [20]. *CCL5* expression positively correlated with *KLRD1* in validation (*r* = 0.78, *P* = 6e−4; Fig. 4b) and discovery cohorts (*r* = 0.74, *P* = 7.3e−9; Additional file 1: Figure S2b). Perforin (*PRF1*) and granzymes (*GZMA*, *GZMB*, *GZMH*) are critical components of cytotoxic granules secreted by NK cells to kill target cells [21]. Expression of each cytotoxic granule gene was positively correlated with *KLRD1* expression at baseline in the validation cohort (0.57 ≤ *r* ≤ 0.62, *P* < 0.03; Fig. 4c–f) and in the discovery cohorts (0.76 ≤ *r* ≤ 0.83, *P* < 3e−9; Additional file 1: Figure S2c–f).

### *KLRD1* expression decreases in the blood and increases in the nasal epithelium after respiratory viral infection

**Fig. 5:**
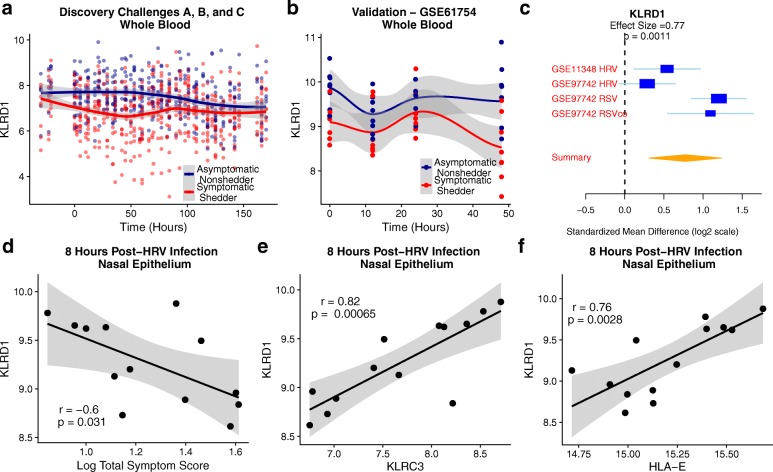
*KLRD1* expression increases in tissue during viral infection and inversely correlates with symptom severity. *KLRD1* expression over the course of viral challenge in **a** discovery challenges A, B, and C and **b** validation cohort GSE61754. **c** Forest plot of *KLRD1* expression in human nasal epithelium infected with human rhinovirus (HRV), respiratory syncytial virus (RSV), or a co-infection of RSV with other pathogens (RSVco) (GSE11348, GSE97742; effect size = 0.77, *P* = 0.001). **d** Correlation between logged total symptom score and *KLRD1* expression in the nasal epithelium 8 h after HRV challenge (GSE11348; *r* = − 0.6, *P* = 0.031). **e**–**f** Correlation between *KLRD1* expression and *KLRC3* (*r* = 0.82, *P* = 0.00065) or *HLA-E* (*r* = 0.76, *P* = 0.0028) expression in the nasal epithelium 8 h after HRV challenge (GSE11348)

*KLRD1* expression further decreased in the blood within the first 48 h of infection in both the discovery (Fig. 5a) and validation (Fig. 5b) cohorts. One possibility for the reduction in *KLRD1* expression in the blood following infection is that *KLRD1*-expressing NK cells are trafficking to the site of infection. Therefore, we sought to examine expression of *KLRD1* in nasal epithelium during acute influenza infection. However, no publicly available studies to our knowledge have profiled human nasal epithelium expression during influenza infection. We have previously described a robust common host immune response to acute respiratory viral infection including influenza, human rhinovirus (HRV), and respiratory syncytial virus (RSV) [13]. Therefore, we utilized a HRV challenge study (GSE11348), and a cohort of children naturally infected with HRV, RSV, or RSV co-infected with other pathogens (RSVco) (GSE97742) [22, 23]. *KLRD1* was expressed at significantly higher levels in virally infected nasal epithelium samples (effect size = 0.77, *P* = 0.0011; Fig. 5c).

In the HRV challenge study (GSE11348), *KLRD1* expression at 8 h post-infection was significantly inversely correlated with symptom severity (*r* = − 0.6, *P* = 0.031; Fig. 5d) similar to influenza challenge studies. We also observed significant positive correlations between *KLRD1* expression and expression of *KLRC3* (*r* = 0.82, *P* = 6.5e−4, Fig. 5e) and *HLA-E* (*r* = 0.76, *p* = 0.0028, Fig. 5f). This data suggests a model where a rapid response by *KLRD1*- and *KLRC3*-expressing NK cells with concurrent upregulation of *HLA-E* by the surrounding tissue may reduce viral infection severity.

### HSPCs decrease in the blood during naturally acquired viral infections

**Fig. 6:**
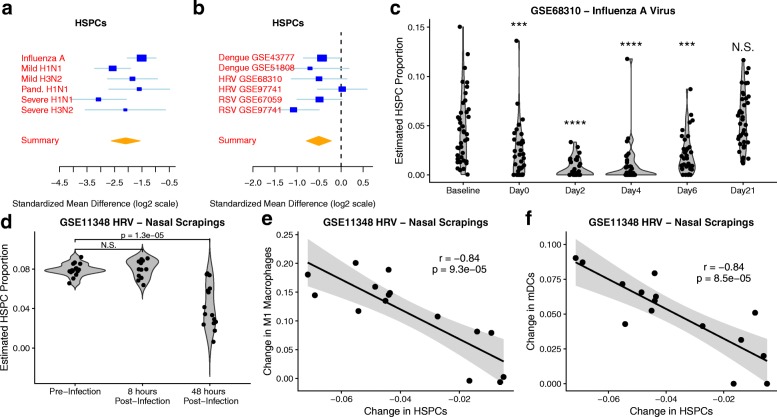
Estimated HSPC proportions decrease in blood and tissue during infection. HSPC proportions were estimated using cell mixture deconvolution in cohorts of acute viral infection. **a** Forest plot indicating the estimated proportion of HSPCs in the blood of individuals acutely infected with influenza compared to controls in cohorts of naturally acquired infection in cohorts obtained from GSE68310 and GSE61821 (summary effect size = − 2.0, *P* < 1e−13). **b** Forest plot indicating the proportions of HSPCs in the blood of individuals acutely infected with dengue, HRV, or RSV compared to controls (summary effect size = 0.5, *P* < 0.001). **c** Time course of HSPC proportions in the blood of individuals with naturally acquired influenza A infection. Baseline indicates a non-infected time point at the beginning of the study. Day 0 is within 48 h of symptom onset. **d** HSPC proportions in nasal scrapings before and after inoculation with rhinovirus (GSE11348). **e**–**f** Correlation between the change in HSPCs between pre-infection and 48 h post-infection and the change in (**e**) M1 macrophages (*r* = − 0.84, *P* = 9.3e−5) and (**f**) mDCs (*r* = − 0.84, *p* = 8.5e−5) between pre-infection and 48 h post-infection. Pand. H1N1: 2009 Pandemic H1N1

Although the difference in HSPC proportions was not statistically significant in validation cohort GSE61754, we observed a trend for higher proportions of HSPCs in symptomatic shedders at baseline (effect size = 0.79, *P* = 0.13; Fig. 2d). It was surprising that HSPCs demonstrated any association with influenza susceptibility, as very little is known about the role of circulating HSPCs in acute infection, particularly in humans. Thus, we investigated changes in HSPC proportions in the blood during acute viral infection. We extended our analysis by performing a meta-analysis of estimated HSPC proportions from naturally acquired influenza cohorts with 236 samples. Individuals with acute influenza infection had consistently lower proportions of HSPCs in the blood than the control time point (summary effect size = − 2.0, *P* < 1e−13; Fig. 6a). To determine whether this was influenza-specific, we performed a meta-analysis of estimated HSPC proportions from naturally occurring non-influenza acute viral cohorts. We included six cohorts of acute dengue, HRV, and RSV infection (339 samples total). We observed a significant decrease in HSPC proportions in acute non-influenza viral infection (effect size = 0.5, *P* < 0.001; Fig. 6b).

We further investigated the dynamics of changes in HSPC proportions in the blood during influenza infection using GSE68310, where individuals provided a baseline healthy sample at the beginning of the flu season, and returned to the clinic within 48 h of symptom onset (day 0) [24]. We observed a significant decrease in HSPC proportions (*p* < 0.0001; Fig. 6c). This decrease in HSPC proportions continued through day 6, and HSPC proportions returned to baseline levels by day 21 (Fig. 6c).

### HSPC proportions decrease in nasal epithelium over the course of rhinovirus challenge and correlate with increases in mDC and M1 macrophage proportions

To study the presence of HSPCs at the site of infection, we examined HSPC proportions from nasal scrapings of human volunteers inoculated with HRV (GSE11348) [22]. HSPC proportions sharply decreased 48 h post-infection in nasal scrapings (*P* = 1.3e−5; Fig. 6d). This decrease could result from trafficking, cell death, or differentiation of HSPCs into mature myeloid cells. To test the hypothesis that HSPCs differentiate into mature cells during viral infection, we correlated the changes in HSPC proportions with the changes of myeloid dendritic cell (mDC) and M1 macrophage proportions between pre-infection and 48 h post-infection. Reductions of HSPC proportions strongly correlated with increased proportions of M1 macrophages (*r* = − 0.84, *p* = 9.3e−5; Fig. 6e) and mDCs (*r* = − 0.84, *P* = 8.5e−5; Fig. 6f), both of which derive from the hematopoietic lineage. This finding is supported by data derived from a cohort of children acutely infected with HRV, RSV, or a co-infection of RSV and other pathogens [23]. We observed in this additional cohort that proportions of HSPCs during acute infection from nasopharyngeal swabs negatively correlated with proportions of M1 macrophages and mDCs (− 0.82 < *r* < − 0.22; 2e−4 < *p* < 0.24; Additional file 1: Figure S3). As the samples with the lowest proportions of HSPCs were the samples with the highest proportions of M1 macrophages and mDCs, this supports a model where HSPCs differentiate into M1 macrophages and mDCs at the site of infection in humans.

## Discussion

Here, we tested a hypothesis that the baseline immune profile prior to influenza inoculation can predict which subject will become infected. We applied cell mixture deconvolution of whole blood transcriptome profiles from four independent influenza challenge studies. Symptomatic shedders had lower NK cell proportions prior to influenza inoculation both in discovery and validation cohorts. Symptomatic shedders had significantly higher HSPC proportions in discovery cohorts with a statistically non-significant trend in the validation cohort. NK cell-associated gene *KLRD1* (CD94) was expressed in the blood at lower levels in symptomatic shedders at baseline in both discovery and validation cohorts, which likely reflects differences in NK cell proportions as *KLRD1* was one of the genes used in immunoStates for estimating proportions of NK cells. Baseline *KLRD1* levels negatively correlated with symptom severity and positively correlated with expression of cytotoxic granule-associated genes. Our results support a model where a rapid response by *KLRD1*-expressing NK cells can lessen severity of or may prevent influenza infection.

NK cells are innate immune cells that can recognize and lyse malignant or virally infected cells [28]. NK cells express a variety of activating and inhibitory receptors that lead to a diverse pool of NK cell phenotypes [26]. *KLRD1* encodes NK cell receptor CD94, which forms a heterodimer with an NKG2 family member, and recognizes HLA-E on target cells [26]. Whether the CD94/NKG2 complex is activating or inhibitory depends on the NKG2 family member involved. The NKG2 family includes inhibitory receptors NKG2A and NKG2B, activating receptor NKG2C, and poorly understood members NKG2E and NKG2H [19, 25]. NKG2E is not expressed on NK cell surface [26], whereas NKG2H is expressed on the surface of a small fraction of human NK cells [27]. By surveying HLA-E levels on target cells, the CD94/NKG2 complex is thought to detect general downregulation of HLA complexes by viruses or cancer [19].

The CD94/NKG2E receptor complex has been shown to be essential for mouse survival when exposed to mousepox [28]. On the other hand, CD94-deficient mice are not susceptible to mouse cytomegalovirus, lymphocytic choriomeningitis virus, vaccinia virus, *Listeria monocytogenes*, or lethal influenza challenge [29, 30]. Importantly, mouse studies have shown that NK cells are harmful upon lethal challenge by promoting excessive lung inflammation, but beneficial during sublethal influenza challenge by promoting the antiviral immune response [21, 31, 32]. These observations in mouse studies further support our results as human challenge studies are most similar to sublethal mouse influenza models.

We observed that *KLRD1* expression in the blood is downregulated in symptomatic shedders at baseline and inversely correlated with symptom severity in a validation cohort. As *KLRD1* expression in the blood reflects NK cell numbers, this suggests that *KLRD1*-expressing NK cells are protective against influenza infection in humans. Furthermore, *KLRD1* expression in the blood correlated with expression of cytotoxic granule-associated genes: *CCL5*, perforin (*PRF1*), and several granzymes (*GZMA*, *GZMB*, *GZMH*). Thus, having a higher proportion of NK cells in the blood may be protective by increasing the proportions of cells with cytotoxic capabilities. Importantly, our analysis focused on transcriptome data. These findings should be further confirmed at the protein level.

The role of *KLRD1* (CD94) in influenza susceptibility cannot be fully understood without considering which NKG2 family members are involved. Although bulk transcriptomic data cannot definitively answer this question, we correlated expression of *KLRD1* with genes encoding NKG2 family members known to form dimers with CD94: *KLRC1*, *KLRC2*, and *KLRC3*. Only *KLRC3*, which encodes two poorly understood isoforms, NKG2E and NKG2H, correlated with *KLRD1* expression at baseline. In mice, the CD94/NKG2E receptor complex is critical for recognizing and clearing mousepox infection [32]. Orbelyan and colleagues have shown that while human NKG2E has functional signaling domains and can form a complex with CD94 and DAP12, CD94/NKG2E is located in the endoplasmic reticulum, not the plasma membrane [30]. Although studies have not yet been published to address the biological relevance of this observation, this raises the possibility that human NKG2E activates NK cells through an unknown intracellular pathway or inhibits NK cells by restricting the amount of DAP12 available at the cell surface. Less is known about isoform NKG2H, which to the best of our knowledge, has not been studied functionally in NK cells. A larger proportion of human T cells express NKG2H on the cell surface than NK cells, and co-crosslinking NKG2H with a NKG2H-specific monoclonal antibody prevents in vitro activation of T cells through an unknown mechanism [27].

These studies have interesting implications for interpreting our finding that *KLRD1* is associated with influenza resistance and *KLRD1* expression positively correlates with *KLRC3* expression in the blood. Individuals with high levels of *KLRD1* (CD94) also have high levels of *KLRC3* (NKG2E or NKG2H) expression. One interpretation of this observation is that there is a higher probability of forming CD94/NKG2E or CD94/NKG2H receptor complexes, based on stoichiometry. These receptor complexes could lead to influenza resistance through unidentified signaling pathways that activate NK cells. However, it is also possible that *KLRC3* expression in the blood simply reflects the number of NK cells present and that *KLRD1*-expressing NK cells are protective against influenza using a mechanism independent of NKG2E or NKG2H signaling.

We also investigated the temporal expression of *KLRD1* during influenza infection. We observed that expression of *KLRD1* decreased in symptomatic shedders 48 h post-influenza inoculation. Therefore, we hypothesized that *KLRD1*-expressing cells rapidly traffic to the site of infection. However, no publicly available dataset has profiled expression from the respiratory tract of human influenza patients. Based on our previous report describing a robust common host immune response to acute respiratory viral infection including influenza, HRV, and RSV, we hypothesized that *KLRD1* expression will change in the nasal epithelium of individuals infected with HRV or RSV [13]. In a HRV challenge study, *KLRD1* expression in nasal scrapings 8 h after infection negatively correlated with symptom severity. *KLRD1* expression also correlated with *KLRC3* (NKG2E or NKG2H) and *HLA-E* expression. As *KLRD1* and *KLRC3* encode CD94/NKG2 receptor complexes, these results support a model where a rapid response by CD94/NKG2*+* NK cells coupled with high expression of HLA-E by infected target cells leads to rapid viral clearance. Increased expression of *KLRD1* and *KLRC3* in nasal epithelium samples and reduced frequency of NK cells in peripheral blood samples are consistent with our hypothesis that the NK cells are actively recruited to the site of infection. Alternatively, it is possible that *KLRD1* and/or *KLRC3* are upregulated on NK cells in lungs of patients with respiratory viral infection or that *KLRD1/KLRC3*-expressing lung NK cells proliferate vigorously at that site.

Our results suggest that *KLRD1* expressing NK cells may be protective against influenza. However, this is undoubtedly only one aspect of influenza susceptibility. Influenza challenge studies routinely exclude individuals with existing antibody titers to the challenge strain, meaning the results may not be directly applicable to individuals with existing B cell memory responses [3, 5]. CD4+ T cell and CD8+ T cell cross-reactive memory responses have also been shown to affect influenza infection susceptibility and severity [7, 8]. Hence, the role of *KLRD1*-expressing NK cells within broader immune system memory must be further studied.

Hematopoietic stem cells (HSCs) have the unique capacity of self-renewal [33]. HSCs differentiate into hematopoietic progenitor cells (HPCs), with varying differentiation capabilities. HSCs and HPCs are difficult to distinguish experimentally and share expression of the surface marker CD34. Thus, we use the term hematopoietic stem and progenitor cells (HSPCs) to encompass both groups. While HSPCs reside primarily in the bone marrow, it has been shown in mice that HSPCs constantly circulate from the bone marrow, through the blood, into the periphery, and finally through the lymphatic system return to the bone marrow [34]. HSPCs express Toll-like receptors (TLR), such as TLR4 and TLR2, enabling them to recognize and respond to infection [35]. In mice, TLR-stimulated HSPCs have been observed to differentiate into myeloid cell types in the periphery, including dendritic cells and macrophages [34].

Our results demonstrate that during acute viral infection, HSPC proportions decrease in the blood, which may reflect emergency myelopoiesis, a process by which hematopoiesis favors the production of myeloid cells at the expense of the lymphoid compartment to replenish myeloid cells during infection [36]. HSPC proportions may decrease in the blood during infection because HSPCs differentiate into myeloid cells in the bone marrow rather than enter circulation. Furthermore, our results demonstrate that HSPC proportions decrease in nasal scrapings upon rhinovirus challenge, and the decrease in HSPCs correlates with an increase in both M1 macrophages and mDCs. This result supports a model where human HSPCs take an active role in the immune response at the site of infection by differentiating into myeloid cells.

We identified a nonsignificant trend of reduced proportions of HSPCs in asymptomatic nonshedders prior to influenza exposure. It is possible that the asymptomatic nonshedders were protected due to a recent inflammatory event that promoted HSPC differentiation into protective M1 macrophages and mDCs. However, the likelihood of a recent inflammatory event in challenge study participants is low as subjects are often excluded from a challenge study for having had a recent flu-like illness [37]. It is also possible that the difference in HSPC proportions is due to normal variation observed in the healthy population. Further studies are needed to identify factors driving HSPC proportion variation.

Our study was limited due to our dependence on publicly available challenge study data. Arguably, the number of samples in the challenge studies used here were low. A post hoc statistical power analysis indicated we had sufficient power to detect NK cell and HSPC immune cell proportion differences [38]. We only included symptomatic shedders and asymptomatic nonshedders in our analysis. It is unclear whether our results are applicable to symptomatic nonshedders and asymptomatic shedders. Participants across all challenge studies were healthy young adults. Our results may not be applicable to children or the elderly and need to be investigated in these groups. Furthermore, we only had access to transcriptomic data. Additional studies should confirm whether symptomatic shedders have lower proportions of NK cells at baseline and whether high expression of *KLRD1* in the blood directly correlates with greater numbers of CD94+ NK cells via flow cytometry.

## Conclusions

In conclusion, we identified *KLRD1*-expressing NK cells as a novel biomarker for influenza susceptibility. We found that *KLRD1* expression correlated with expression of cytotoxic granule-associated genes, suggesting that higher *KLRD1* expression may correlate with increased proportions of cytotoxic immune cells. We showed that higher *KLRD1* expression in the nasal epithelium 8 h after HRV infection was associated with reduced symptom severity. Our results imply that an early response by *KLRD1*-expressing NK cells may reduce symptom severity and possibly prevent influenza infection entirely. The seasonal influenza vaccine has already been shown to stimulate memory-like NK cell responses in humans [39]. Future vaccination strategies may benefit from not only targeting B cells and T cells but also enhancing *KLRD1*-expressing NK cell responses.

## Additional file

Additional file 1: **Table S1.** Figures S1–S3; Supplemental methods. (PDF 360 kb)

## Abbreviations

FDR: False discovery rate
GEO: Gene Expression Omnibus
HPC: Hematopoietic progenitor cell
HRV: Human rhinovirus
HSC: Hematopoietic stem cell
HSPC: Hematopoietic stem and progenitor cell
mDC: Myeloid dendritic cell
NCBI: National Center for Biotechnology Information
NK: Natural Killer
RSV: Respiratory syncytial virus
SEM: Standard error of mean

## References

[1] CDC: Disease Burden of Influenza [https://www.cdc.gov/flu/about/disease/burden.htm].

[2] Zaas AK, Chen M, Varkey J, Veldman T, Hero AO, Lucas J, Huang Y, Turner R, Gilbert A, Lambkin-Williams R (2009). Gene expression signatures diagnose influenza and other symptomatic respiratory viral infections in humans. Cell Host Microbe.

[3] Woods CW, McClain MT, Chen M, Zaas AK, Nicholson BP, Varkey J, Veldman T, Kingsmore SF, Huang Y, Lambkin-Williams R (2013). A host transcriptional signature for presymptomatic detection of infection in humans exposed to influenza H1N1 or H3N2. PLoS One.

[4] Muller J, Parizotto E, Antrobus R, Francis J, Bunce C, Stranks A, Nichols M, McClain M, Hill AVS, Ramasamy A (2017). Development of an objective gene expression panel as an alternative to self-reported symptom scores in human influenza challenge trials. J Transl Med.

[5] Davenport EE, Antrobus RD, Lillie PJ, Gilbert S, Knight JC (2015). Transcriptomic profiling facilitates classification of response to influenza challenge. J Mol (Berlin).

[6] Liu TY, Burke T, Park LP, Woods CW, Zaas AK, Ginsburg GS, Hero AO (2016). An individualized predictor of health and disease using paired reference and target samples. BMC bioinformatics.

[7] Wilkinson TM, Li CK, Chui CS, Huang AK, Perkins M, Liebner JC, Lambkin-Williams R, Gilbert A, Oxford J, Nicholas B (2012). Preexisting influenza-specific CD4+ T cells correlate with disease protection against influenza challenge in humans. Nat Med.

[8] Sridhar S, Begom S, Bermingham A, Hoschler K, Adamson W, Carman W, Bean T, Barclay W, Deeks JJ, Lalvani A (2013). Cellular immune correlates of protection against symptomatic pandemic influenza. Nat Med.

[9] Shen-Orr SS, Gaujoux R (2013). Computational deconvolution: extracting cell type-specific information from heterogeneous samples. Curr Opin Immunol.

[10] Inkeles MS, Teles RM, Pouldar D, Andrade PR, Madigan CA, Lopez D, Ambrose M, Noursadeghi M, Sarno EN, Rea TH (2016). Cell-type deconvolution with immune pathways identifies gene networks of host defense and immunopathology in leprosy. JCI insight.

[11] Gentles AJ, Newman AM, Liu CL, Bratman SV, Feng W, Kim D, Nair VS, Xu Y, Khuong A, Hoang CD (2015). The prognostic landscape of genes and infiltrating immune cells across human cancers. Nat Med.

[12] Vallania F, Tam A, Lofgren S, Schaffert S, Azad TD, Bongen E, Alsup M, Alonso M, Davis M, Engleman E (2017). Leveraging heterogeneity across multiple data sets increases accuracy of cell-mixture deconvolution and reduces biological and technical biases. bioRxiv.

[13] Andres-Terre M, McGuire Helen M, Pouliot Y, Bongen E, Sweeney Timothy E, Tato Cristina M, Khatri P (2015). Integrated, multi-cohort analysis identifies conserved transcriptional signatures across multiple respiratory viruses. Immunity.

[14] Edgar R, Domrachev M, Lash AE (2002). Gene Expression Omnibus: NCBI gene expression and hybridization array data repository. Nucleic Acids Res.

[15] Haynes WA, Vallania F, Liu C, Bongen E, Tomczak A, Andres-Terre M, Lofgren S, Tam A, Deisseroth CA, Li MD (2017). Empowering multi-cohort gene expression analysis to increase reproducibility. Pac Symp Biocomput.

[16] Lofgren S, Hinchcliff M, Carns M, Wood T, Aren K, Arroyo E, Cheung P, Kuo A, Valenzuela A, Haemel A (2016). Integrated, multicohort analysis of systemic sclerosis identifies robust transcriptional signature of disease severity. JCI insight.

[17] Sweeney TE, Haynes WA, Vallania F, Ioannidis JP, Khatri P (2017). Methods to increase reproducibility in differential gene expression via meta-analysis. Nucleic Acids Res.

[18] Benjamini Y, Hochberg Y (1995). Controlling the false discovery rate: a practical and powerful approach to multiple testing. J R Statist Soc.

[19] Martinet L, Smyth MJ (2015). Balancing natural killer cell activation through paired receptors. Nat Rev Immunol.

[20] Kumar D, Hosse J, von Toerne C, Noessner E, Nelson PJ (2009). JNK MAPK pathway regulates constitutive transcription of CCL5 by human NK cells through SP1. J Immunol.

[21] Lam VC, Lanier LL (2017). NK cells in host responses to viral infections. Curr Opin Immunol.

[22] Proud D, Turner RB, Winther B, Wiehler S, Tiesman JP, Reichling TD, Juhlin KD, Fulmer AW, Ho BY, Walanski AA (2008). Gene expression profiles during in vivo human rhinovirus infection: insights into the host response. Am J Respir Crit Care Med.

[23] Do LAH, Pellet J, van Doorn HR, Tran AT, Nguyen BH, Tran TTL, Tran QH, Vo QB, Tran Dac NA, Trinh HN (2017). Host transcription profile in nasal epithelium and whole blood of hospitalized children under 2 years of age with respiratory syncytial virus infection. J Infect Dis.

[24] Zhai Y, Franco LM, Atmar RL, Quarles JM, Arden N, Bucasas KL, Wells JM, Nino D, Wang X, Zapata GE (2015). Host transcriptional response to influenza and other acute respiratory viral infections—a prospective cohort study. PLoS Pathog.

[25] Lieto LD, Maasho K, West D, Borrego F, Coligan JE (2006). The human CD94 gene encodes multiple, expressible transcripts including a new partner of NKG2A/B. Genes Immun.

[26] Orbelyan GA, Tang F, Sally B, Solus J, Meresse B, Ciszewski C, Grenier JC, Barreiro LB, Lanier LL, Jabri B (2014). Human NKG2E is expressed and forms an intracytoplasmic complex with CD94 and DAP12. J Immunol.

[27] Dukovska D, Fernandez-Soto D, Vales-Gomez M, Reyburn HT (2018). NKG2H-expressing T cells negatively regulate immune responses. Front Immunol.

[28] Fang M, Orr MT, Spee P, Egebjerg T, Lanier LL, Sigal LJ (2011). CD94 is essential for NK cell-mediated resistance to a lethal viral disease. Immunity.

[29] Shin DL, Pandey AK, Ziebarth JD, Mulligan MK, Williams RW, Geffers R, Hatesuer B, Schughart K, Wilk E (2014). Segregation of a spontaneous Klrd1 (CD94) mutation in DBA/2 mouse substrains. G3 (Bethesda).

[30] Orr MT, Wu J, Fang M, Sigal LJ, Spee P, Egebjerg T, Dissen E, Fossum S, Phillips JH, Lanier LL (2010). Development and function of CD94-deficient natural killer cells. PLoS One.

[31] Abdul-Careem MF, Mian MF, Yue G, Gillgrass A, Chenoweth MJ, Barra NG, Chew MV, Chan T, Al-Garawi AA, Jordana M (2012). Critical role of natural killer cells in lung immunopathology during influenza infection in mice. J Infect Dis.

[32] Ge MQ, Ho AW, Tang Y, Wong KH, Chua BY, Gasser S, Kemeny DM (2012). NK cells regulate CD8+ T cell priming and dendritic cell migration during influenza A infection by IFN-gamma and perforin-dependent mechanisms. J Immunol.

[33] Mazo IB, Massberg S, von Andrian UH (2011). Hematopoietic stem and progenitor cell trafficking. Trends Immunol.

[34] Massberg S, Schaerli P, Knezevic-Maramica I, Kollnberger M, Tubo N, Moseman EA, Huff IV, Junt T, Wagers AJ, Mazo IB (2007). Immunosurveillance by hematopoietic progenitor cells trafficking through blood, lymph, and peripheral tissues. Cell.

[35] Nagai Y, Garrett KP, Ohta S, Bahrun U, Kouro T, Akira S, Takatsu K, Kincade PW (2006). Toll-like receptors on hematopoietic progenitor cells stimulate innate immune system replenishment. Immunity.

[36] Takizawa H, Boettcher S, Manz MG (2012). Demand-adapted regulation of early hematopoiesis in infection and inflammation. Blood.

[37] Lillie PJ, Berthoud TK, Powell TJ, Lambe T, Mullarkey C, Spencer AJ, Hamill M, Peng Y, Blais ME, Duncan CJ (2012). Preliminary assessment of the efficacy of a T-cell-based influenza vaccine, MVA-NP+M1, in humans. Clin Infect Dis.

[38] Valentine JC, Pigott TD, Rothstein HR (2010). How many studies do you need?:a primer on statistical power for meta-analysis. J Educ Behav Stat.

[39] Dou Y, Fu B, Sun R, Li W, Hu W, Tian Z, Wei H (2015). Influenza vaccine induces intracellular immune memory of human NK cells. PLoS One.

[40] Hoang LT, Tolfvenstam T, Ooi EE, Khor CC, Naim AN, Ho EX, Ong SH, Wertheim HF, Fox A, Van Vinh Nguyen C (2014). Patient-based transcriptome-wide analysis identify interferon and ubiquination pathways as potential predictors of influenza A disease severity. PLoS One.

[41] Sun P, Garcia J, Comach G, Vahey MT, Wang Z, Forshey BM, Morrison AC, Sierra G, Bazan I, Rocha C (2013). Sequential waves of gene expression in patients with clinically defined dengue illnesses reveal subtle disease phases and predict disease severity. PLoS Negl Trop Dis.

[42] Kwissa M, Nakaya HI, Onlamoon N, Wrammert J, Villinger F, Perng GC, Yoksan S, Pattanapanyasat K, Chokephaibulkit K, Ahmed R (2014). Dengue virus infection induces expansion of a CD14(+)CD16(+) monocyte population that stimulates plasmablast differentiation. Cell Host Microbe.

[43] Heinonen S, Jartti T, Garcia C, Oliva S, Smitherman C, Anguiano E, de Steenhuijsen Piters WA, Vuorinen T, Ruuskanen O, Dimo B (2016). Rhinovirus detection in symptomatic and asymptomatic children: value of host transcriptome analysis. Am J Respir Crit Care Med.

