# Supplementary material for: KLRD1-expressing natural killer cells predict influenza susceptibility
Source: Genome Med. 2018 Jun 14;10:45. doi: 10.1186/s13073-018-0554-1 (PMC6001128; doi:10.1186/s13073-018-0554-1)
Supplement: Supplementary file 1 — Table S1. Figures S1–S3; Supplemental methods. (PDF 360 kb) [file 13073_2018_554_MOESM1_ESM.pdf]

Additional file 1: Supplemental materials for the paper *KLRD1* expressing natural killer cells predict influenza susceptibility

Erika Bongen[1,2], Francesco Vallania [1,3], Paul J Utz [1,2,4], Purvesh Khatri[1,2,3]*

[1]Institute for Immunity, Transplantation and Infection, Stanford University School of Medicine, Stanford, CA 94305, USA

[2]Program in Immunology, Stanford University School of Medicine, Stanford, CA, USA

[3]Department of Medicine, Division of Biomedical Informatics Research, Stanford University School of Medicine, Stanford, CA 94305, USA

[4]Department of Medicine, Division of Immunology and Rheumatology, Stanford University School of Medicine, Stanford, CA 94305, USA

*Corresponding author: Purvesh Khatri

Table S1: Cell types not detected by cell mixture deconvolution by cohort

	GSE73072 Challenge A	GSE73072 Challenge B	GSE73072 Challenge C	GSE61754
**CD56dim Natural Killer cells**	N.D.	N.D.	N.D.	N.D.
**Gamma-delta T cell**	N.D.	N.D.	N.D.	N.D.
**M0 Macrophage**	N.D.	N.D.	N.D.	N.D.
**Myeloid Dendritic Cells**	N.D.	N.D.	N.D.	N.D.
**Plasma Cells**	N.D.	N.D.	Detected	N.D.
**Memory B cells**	N.D.	N.D.	N.D.	Detected
**Basophils**	N.D.	N.D.	N.D.	Detected
**Mast Cells**	Detected	Detected	Detected	N.D.

N.D. - Not Detected

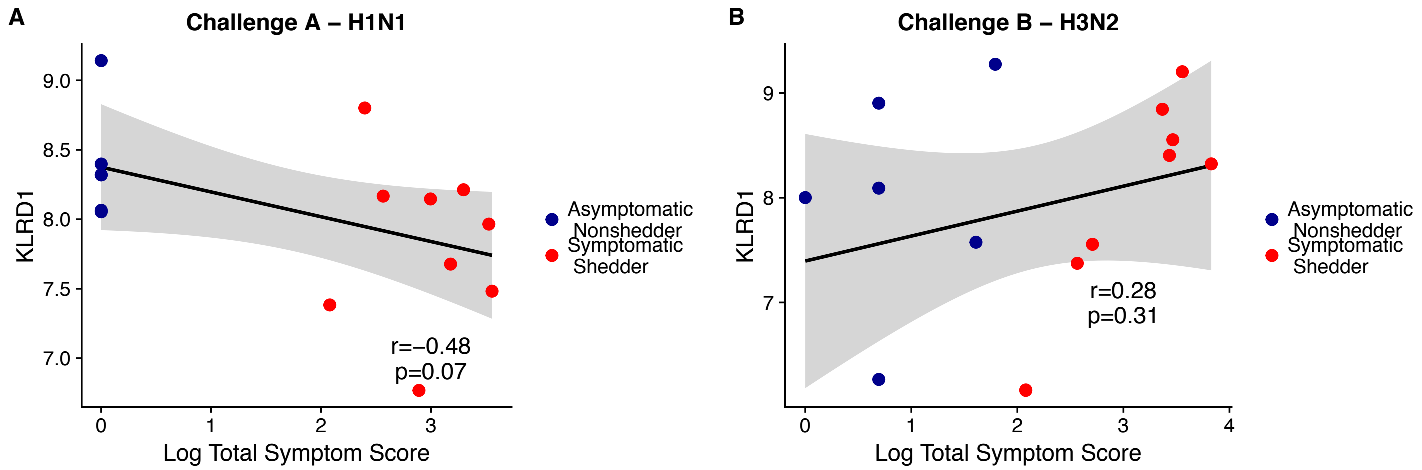

Figure S1: Correlation between baseline KLRD1 expression and logged total symptom score in discovery cohorts Challenge A and Challenge B. (A) Correlation between baseline KLRD1 expression and log total symptom score in Challenge A (r=-0.48, P=0.07). (B) Correlation between baseline KLRD1 expression and log total symptom score in Challenge B (r=0.28, P=0.31).

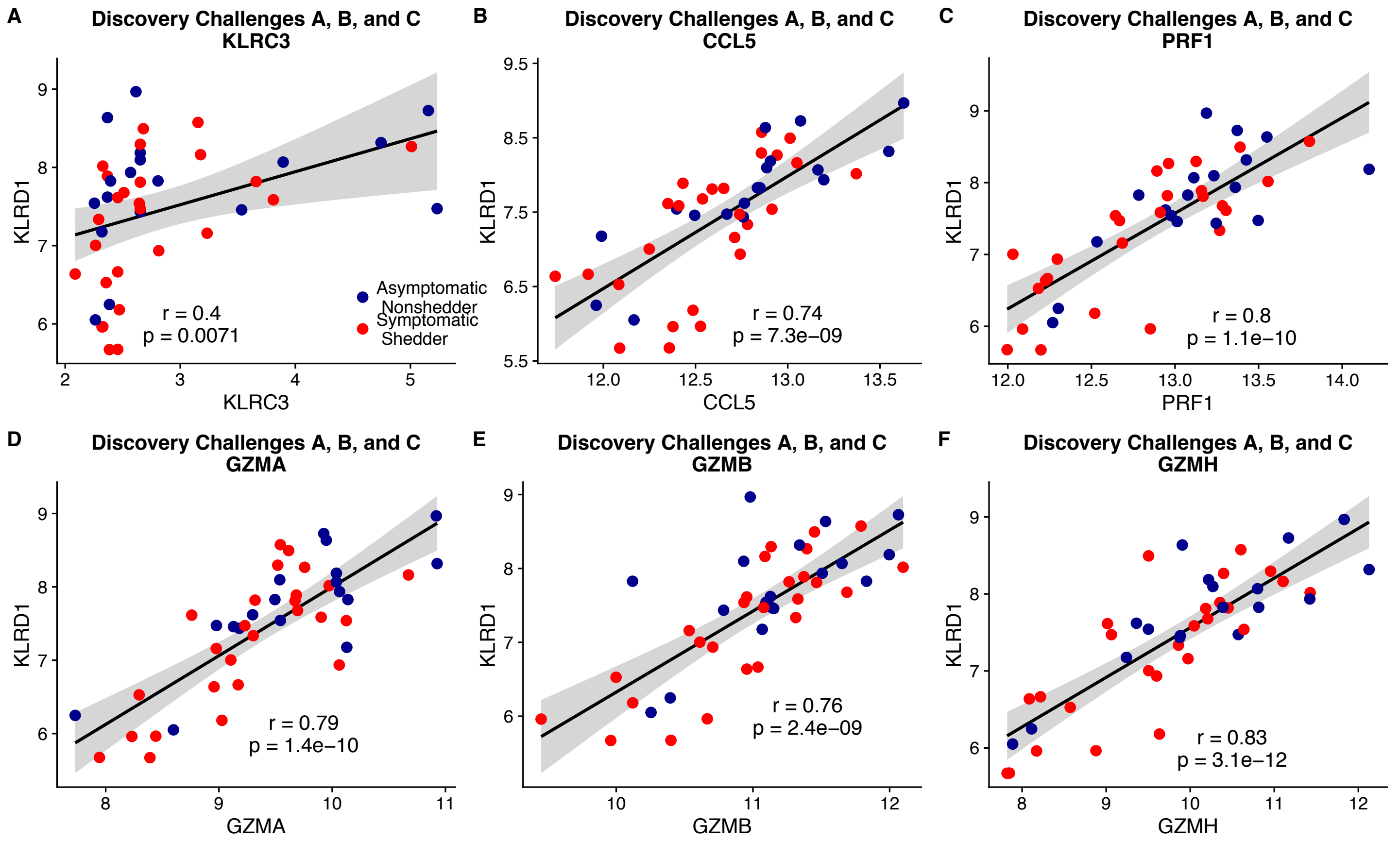

Figure S2: ***KLRD1*** **correlates with** ***KLRC3*** **and cytotoxic granule-associated genes at baseline in discovery cohorts Challenge A, B, and C.** Gene expression from ComBat co-normalized discovery cohorts, Challenge A, B, and C prior to infection demonstrating correlations between *KLRD1* expression and *KLRC3* (A, r=0.4, P=0.0071), and B-F) cytotoxic granule associated genes: *CCL5* (B, r=0.74, P=7.3e-9), perforin (C, *PRF1*, r=0.8, P=1.1e-10), granzyme A (D, *GZMA*, r=0.79, P=1.4e-10), granzyme B (E, *GZMB*, r=0.76, P=2.4e-9), and granzyme H (F, *GZMH*, r=0.83, P=3.1e-12).

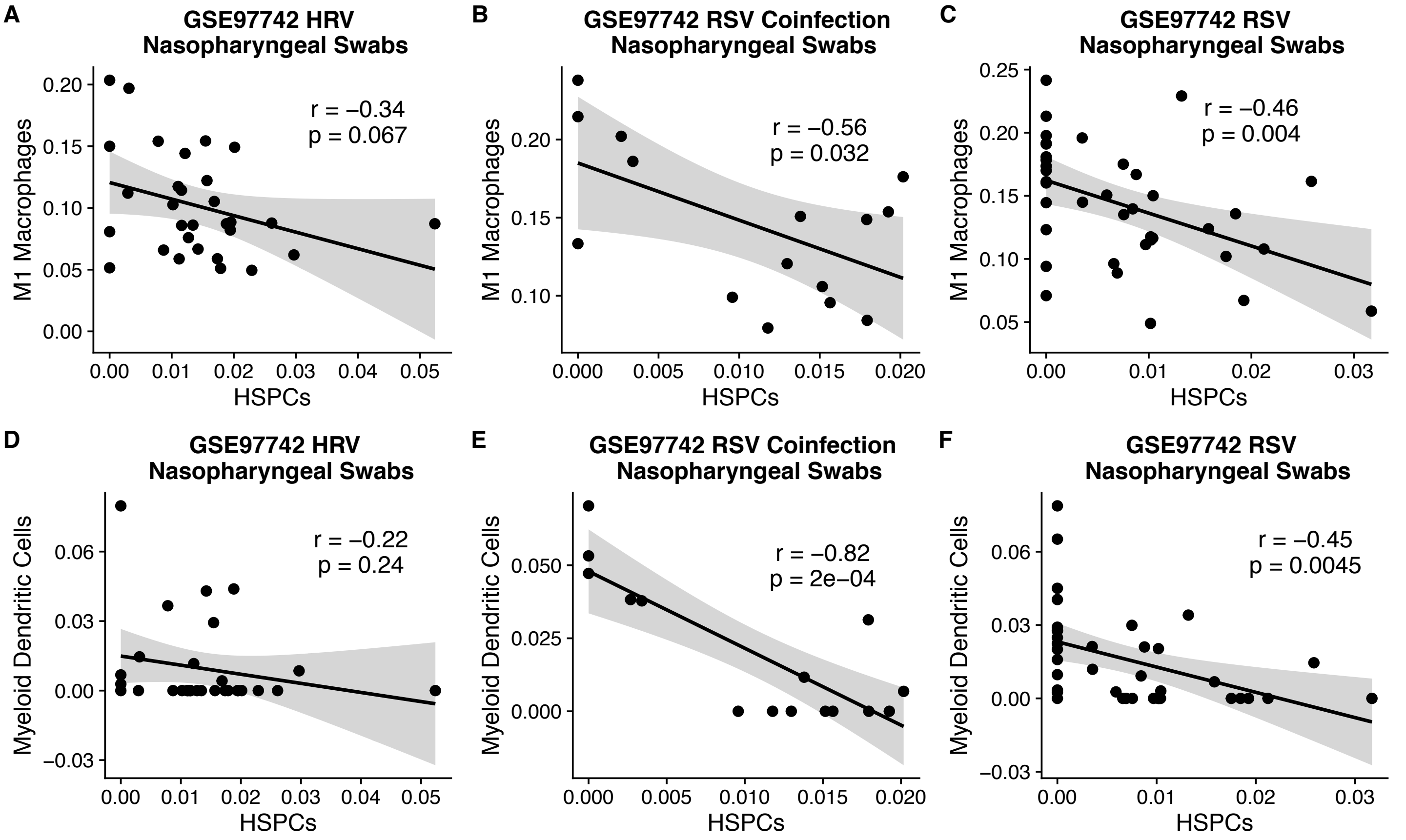

Figure S3: Inverse correlations between estimated HSPCs, M1 macrophages, and mDCs from nasopharyngeal swabs of acutely infected children. (A-C) Correlations between estimated proportions of HSPCs and M1 macrophages from nasopharyngeal swabs of children acutely infected with A) HRV (r=-0.34, P=0.067), B) RSV coinfected with other pathogens (r=-0.56, P=0.032), and C) RSV alone (r=-0.46, P=0.004), estimated using cell mixture deconvolution (D-F) Correlations between estimated proportions of HSPCs and myeloid dendritic cells from nasopharyngeal swabs of children acutely infected with D) HRV (r=-0.22, p=0.24), E) RSV coinfected with other pathogens (r=-0.82, p=2e-4), and C) RSV alone (r=-0.45, p=0.0045), estimated using cell mixture deconvolution. HSPC: hematopoietic stem and progenitor cells. RSV**:** Respiratory Syncytial Virus HRV**:** Human Rhinovirus

Supplemental Methods

Data Collection and Preprocessing

We identified 4 influenza challenge studies consisting of 52 whole blood samples the NCBI database Gene Expression Omnibus (GEO) (**Table 1**) [1]. We supplemented the influenza challenge cohorts with 7 acute viral infection studies consisting of 16 cohorts of 771 whole blood, PBMC, and nasal epithelium samples also from GEO (**Table 2**) [1]. We excluded challenge studies with less than 5 asymptomatic-nonshedders or 5 symptomatic-shedders. We used phenotypic labels as reported by the original authors. All datasets used were publicly available as described below.

All datasets were downloaded using the MetaIntegrator R Package [2]. Unless otherwise specified, we used gene expression data that had been preprocessed by the original authors, after verifying normalization and log2-transformation.

Cohort Summaries

Liu et al. 2016 Cohorts (GSE73072)

The Liu et al. 2016 viral challenge cohorts were obtained from GEO (GSE73072) [3]. Three cohorts within GSE73072 fit our inclusion criteria as influenza challenge studies with at least 5 asymptomatic-nonshedders and 5 symptomatic-shedders. These cohorts (which we renamed for clarity) were DEE2 (Challenge B), DEE3 (Challenge A), and DEE5 (Challenge C). To maintain the heterogeneity of the cohorts, the .CEL files were downloaded from GEO, and each cohort was separately RMA-normalized and log-2 transformed. Total symptom scores were obtained for DEE2 (Challenge B) and DEE3 (Challenge A) from an earlier published version of those cohorts (GSE52428).

Davenport et al. 2015 cohort (GSE61754)

The Davenport et al. 2015 cohort (GSE61754) contained gene expression microarray data from whole blood of healthy individuals challenged with influenza H3N2 [4]. We utilized the dataset as preprocessed by the original authors, after verifying normalization and log2-transformation. The symptoms scores and demographic information were obtained through correspondence with the authors.

Proud et al. 2008 cohort (GSE11348)

The Proud et al. 2008 contained gene expression microarray data from the nasal scrapings of individuals experimentally infected with Human Rhinovirus (HRV-16) [5]. This cohort also contained sham-infected individuals, which we removed from this study. We downloaded the cohort from GEO (GSE11348) and utilized the data as preprocessed by the original authors, after verifying normalization and log2-transformation.

Do et al. 2018 cohort (GSE97742 and GSE97741)

The Do et al. 2018 cohort contained gene expression microarray data from nasopharyngeal swabs (GSE97742) or whole blood (GSE97741) of children < 2 years old hospitalized with lower respiratory tract infection at admission to hospital (acute infection) and discharge [6]. We downloaded the cohort from GEO (GSE97742, GSE97741) and utilized the data as preprocessed by the original authors, after verifying normalization and log2-transformation. We separated GSE97742 and GSE97741 each into three cohorts by virus: Human Rhinovirus (HRV), Respiratory Syncytial Virus (RSV), and RSV coinfected with other pathogens (RSVco).

Hoang et al. 2014 cohort (GSE61821)

GSE61821 (Hoang et al. 2014) contained gene expression microarray data from whole blood of individuals with natural influenza infection between the ages of 5-73 years old [7]. We downloaded the cohort from GEO (GSE61821) and utilized the data as preprocessed by the original authors, after verifying normalization and

log2-transformation. We separated GSE61821 into five cohorts according to virus and infection severity: Mild H1N1, Mild H3N2, Severe H1N1, Severe H3N2, and Pandemic H1N1 (Pand. H1N1). We defined severe infection as needing hospitalization, and thus both patients labeled as "moderate" and patients labeled as "severe" in GSE61821 are categorized as "severe" in our work. Due to small sample size, the Pandemic H1N1 cohort was not separated into "mild" and "severe".

Zhai et al. 2015 cohorts (GSE68310)

GSE68310 (Zhai et al. 2015) contained gene expression microarray data from whole blood of adults with naturally acquired respiratory infections [8]. Individuals in GSE68310 were first profiled at a baseline healthy time point prior to viral infection and then returned for additional transcriptional profiling within 48 hours of symptom onset (study Day 0) of a naturally acquired respiratory viral infection. We downloaded the cohort from GEO (GSE61821) and utilized the data as preprocessed by the original authors, after verifying normalization and log2-transformation. We separated GSE68310 into two cohorts based on virus: Influenza A and Human Rhinovirus (HRV). Individuals infected with other viruses were excluded due to low sample size.

Sun et al. 2013 cohort (GSE43777)

The Sun et al. 2013 cohort (GSE43777) contained gene expression microarray data from peripheral blood mononuclear cells (PBMCs) of humans during the acute, late acute, and convalescent phases of dengue infection [9]. We downloaded the normalized and log2-transformed data from GEO (GSE43777). Individuals with dengue hemorrhagic fever were excluded. Samples of late acute infection were excluded. GSE43777 was profiled on two microarray platforms, but only samples profiled on Affymetrix Human HG-Focus Target Array (GPL201) were used due to its larger sample size.

Kwissa et al. 2014 cohort (GSE51808)

The Kwissa et al. 2014 cohort (GSE51808) contained gene expression microarray data from whole blood of individuals infected with dengue and healthy controls [10]. We downloaded the normalized and log-2

transformed data from GEO (GSE51808). Individuals with dengue hemorrhagic fever were excluded. Only samples from acute dengue infection or healthy controls were included.

Heinonen et al. 2016 cohort (GSE67059)

The Heinonen et al. 2016 cohort (GSE67059) contained gene expression microarray data from whole blood of children < 2 years old symptomatically infected with HRV, asymptomatically infected with HRV, and healthy controls [11]. We log2-transformed the normalized data obtained from GEO (GSE67059). Asymptomatically infected individuals were excluded.

References

1. Edgar R, Domrachev M, Lash AE: **Gene Expression Omnibus: NCBI gene expression and hybridization array data repository**. *Nucleic Acids Res* 2002, **30**(1):207-210.
2. Haynes WA, Vallania F, Liu C, Bongen E, Tomczak A, Andres-Terre M, Lofgren S, Tam A, Deisseroth CA, Li MD *et al*: **EMPOWERING MULTI-COHORT GENE EXPRESSION ANALYSIS TO INCREASE REPRODUCIBILITY**. *Pacific Symposium on Biocomputing Pacific Symposium on Biocomputing* 2017, **22**:144-153.
3. Liu TY, Burke T, Park LP, Woods CW, Zaas AK, Ginsburg GS, Hero AO: **An individualized predictor of health and disease using paired reference and target samples**. *BMC bioinformatics* 2016, **17**:47.
4. Davenport EE, Antrobus RD, Lillie PJ, Gilbert S, Knight JC: **Transcriptomic profiling facilitates classification of response to influenza challenge**. *Journal of molecular medicine (Berlin, Germany)* 2015, **93**(1):105-114.
5. Proud D, Turner RB, Winther B, Wiehler S, Tiesman JP, Reichling TD, Juhlin KD, Fulmer AW, Ho BY, Walanski AA *et al*: **Gene expression profiles during in vivo human rhinovirus infection: insights into the host response**. *American journal of respiratory and critical care medicine* 2008, **178**(9):962-968.
6. Do LAH, Pellet J, van Doorn HR, Tran AT, Nguyen BH, Tran TTL, Tran QH, Vo QB, Tran Dac NA, Trinh HN *et al*: **Host Transcription Profile in Nasal Epithelium and Whole Blood of Hospitalized Children Under 2 Years of Age With Respiratory Syncytial Virus Infection**. *The Journal of infectious diseases* 2017, **217**(1):134-146.
7. Hoang LT, Tolfvenstam T, Ooi EE, Khor CC, Naim AN, Ho EX, Ong SH, Wertheim HF, Fox A, Van Vinh Nguyen C *et al*: **Patient-based transcriptome-wide analysis identify interferon and ubiquination pathways as potential predictors of influenza A disease severity**. *PloS one* 2014, **9**(11):e111640.
8. Zhai Y, Franco LM, Atmar RL, Quarles JM, Arden N, Bucasas KL, Wells JM, Nino D, Wang X, Zapata GE *et al*: **Host Transcriptional Response to Influenza and Other Acute Respiratory Viral Infections--A Prospective Cohort Study**. *PLoS pathogens* 2015, **11**(6):e1004869.
9. Sun P, Garcia J, Comach G, Vahey MT, Wang Z, Forshey BM, Morrison AC, Sierra G, Bazan I, Rocha C *et al*: **Sequential waves of gene expression in patients with clinically defined dengue illnesses**

reveal subtle disease phases and predict disease severity. *PLoS neglected tropical diseases* 2013, **7**(7):e2298.

10. Kwissa M, Nakaya HI, Onlamoon N, Wrammert J, Villinger F, Perng GC, Yoksan S, Pattanapanyasat K, Chokephaibulkit K, Ahmed R *et al*: **Dengue virus infection induces expansion of a CD14(+)CD16(+) monocyte population that stimulates plasmablast differentiation**. *Cell host & microbe* 2014, **16**(1):115-127.
11. Heinonen S, Jartti T, Garcia C, Oliva S, Smitherman C, Anguiano E, de Steenhuijsen Piters WA, Vuorinen T, Ruuskanen O, Dimo B *et al*: **Rhinovirus Detection in Symptomatic and Asymptomatic Children: Value of Host Transcriptome Analysis**. *American journal of respiratory and critical care medicine* 2016, **193**(7):772-782.
